# Vitamin D Deficiency and Clinical Outcomes in Critically Ill Pediatric Patients: A Systematic Review and Meta-Analysis

**DOI:** 10.1210/jendso/bvaf053

**Published:** 2025-04-02

**Authors:** Chai-Hoon Nowel Tan, Bernita Yeo, Rashida Farhad Vasanwala, Rehena Sultana, Jan Hau Lee, Daniel Chan

**Affiliations:** Department of Pediatrics, KK Women's and Children's Hospital, Singapore 229899, Singapore; Department of Pediatrics, KK Women's and Children's Hospital, Singapore 229899, Singapore; Endocrinology Service, Department of Pediatric Medicine, KK Women's and Children's Hospital, Singapore 229899, Singapore; Pediatrics Academic Clinical Programme, Duke-NUS Medical School, Singapore 169857, Singapore; Centre for Quantitative Medicine, Duke-NUS Medical School, Singapore 169857, Singapore; Pediatrics Academic Clinical Programme, Duke-NUS Medical School, Singapore 169857, Singapore; Pediatric Intensive Care, Department of Pediatric Subspecialties, KK Women's and Children's Hospital, Singapore 229899, Singapore; Endocrinology Service, Department of Pediatric Medicine, KK Women's and Children's Hospital, Singapore 229899, Singapore; Pediatrics Academic Clinical Programme, Duke-NUS Medical School, Singapore 169857, Singapore

**Keywords:** vitamin D, pediatric intensive care unit, critical care outcomes

## Abstract

**Context:**

Vitamin D deficiency (VDD) is common in paediatric populations, and its relationship with critical care outcomes warrants further investigation.

**Objective:**

The aim is to examine the association between VDD and clinical outcomes in children admitted to the Pediatric Intensive Care Unit (PICU).

**Methods:**

This systematic review and meta-analysis investigated the impact of VDD on clinical outcomes in PICU patients. A comprehensive search of Embase, Web of Science, PubMed, and Cochrane databases was conducted. Our primary outcomes were mortality and sepsis incidence, while secondary outcomes included length of stay (LOS), need for inotropic support, and need for and duration of mechanical ventilation. Eligible studies included infants and children aged 1 month to 18 years admitted to the PICU, with baseline 25-hydroxyvitamin D levels measured on admission. Two independent reviewers screened studies, extracted data, and assessed quality. Pooled estimates were obtained using a random-effects model.

**Results:**

Out of 2298 screened studies, 27 met the inclusion criteria, comprising 4682 patients. VDD was defined as 25-hydroxyvitamin D levels <20 ng/mL and <30 ng/mL in 22 and 5 studies, respectively. VDD was associated with increased mortality (odds ratio [OR] 2.05, 95% CI 1.21-3.48) and a greater need for inotropic support (OR 2.02, 95% CI 1.43-2.85) than children with vitamin D sufficiency (VDS). No differences were observed between VDD and VDS groups in terms of sepsis incidence postadmission, LOS, or the need for and duration of mechanical ventilation.

**Conclusion:**

VDD in critically ill pediatric patients was associated with increased mortality and higher need for inotropic support. Further research is warranted to evaluate the potential benefits of vitamin D supplementation in this high-risk population.

Vitamin D deficiency (VDD), commonly reported in critically ill pediatric patients [1], is associated with adverse clinical outcomes [2], including a higher incidence of septic shock [3] and increased risks of requiring inotropic support and mechanical ventilation (MV) [4]. While traditionally recognized for its role in calcium homeostasis, vitamin D is increasingly acknowledged as a key modulator of the immune system [5]. In the pediatric population, its anti-inflammatory and antimicrobial properties are particularly significant, as vitamin D regulates leukocyte activity and cytokine production [6]. Additionally, vitamin D plays a crucial role in muscle protein synthesis and bone strength [7], with implications for rehabilitation outcomes [8]. Immune dysregulation, partly due to VDD, is frequently noted in up to 50% of critically ill patients [9, 10]. Preclinical studies have demonstrated that vitamin D exerts substantial immunomodulatory effects on both the innate and adaptive immune systems [11]. As a result, vitamin D supplementation has been considered in Pediatric Intensive Care Unit (PICU) settings to potentially improve clinical outcomes [12].

In adult patients, the impact of VDD in critical care settings is well described. A recent randomized controlled study noted that while high-dose vitamin D supplementation did not significantly reduce hospital length of stay (LOS), it was associated with lower ICU mortality in a subgroup of patients with severe VDD [13]. Similarly, a systematic review and meta-analysis involving 2449 critically ill adult patients demonstrated reduced mortality with vitamin D administration [14].

In contrast, data regarding the role of vitamin D in pediatric critical care remain inconsistent. Few randomized controlled studies have been conducted, and findings have varied. For instance, Xie et al [15] observed that vitamin D supplementation in 416 children admitted to a PICU effectively reduced *Candida* infections. Additionally, systematic reviews have identified associations between VDD, increased mortality, and greater illness severity in critically ill children [16, 17]. However, some observational studies have reported no clear association between VDD and illness severity [18], while others highlighted uncertainties in the relationship between vitamin D status and critical care outcomes [19].

With a high prevalence of pediatric VDD reported worldwide [20] and its potential role in immunoregulation, this systematic review and meta-analysis aims to consolidate the existing evidence on VDD and its association with clinical outcomes in critically ill children. The findings could provide valuable insights to guide future research and considerations for vitamin D supplementation in pediatric critical care.

## Materials and Methods

### Search Strategy

This review was registered in PROSPERO (CRD42023481239) on December 5, 2023. A systematic search was performed across 4 databases (PubMed, EMBASE, Web of Science, and the Cochrane Library) for articles published from inception until September 4, 2024. Additionally, reference lists of relevant studies were manually searched to identify further eligible articles. No timeline or language restrictions were applied in the selection of studies. The complete search strategy, including the Medical Subject Headings (MeSH) terms used, is provided (Table 1). Eligible studies included randomized controlled trials (RCTs), as well as observational studies such as prospective and retrospective cohort studies, and case–control studies.

**Table 1.** Search strings

Database	Search strings
Publication date: January 1, 2013-October 7, 2023	
(1) Medline (PubMed)	((“Vitamin D” [Mesh]) OR (Vitamin D [Title/Abstract] OR Cholecalciferol [Title/Abstract] OR Hydroxycholecalciferols [Title/Abstract] OR Calcifediol [Title/Abstract] OR Calcitriol [Title/Abstract] OR Dihydroxycholecalciferols [Title/Abstract] OR 25-Hydroxyvitamin D 2 [Title/Abstract] OR Ergocalciferols [Title/Abstract])) AND ((“Intensive Care Units” [Mesh] OR “Critical Care” [Mesh]) OR (Intensive Care Units [Title/Abstract] OR Intensive Care Units, Pediatric [Title/Abstract] OR Intensive Care Units, Neonatal [Title/Abstract] OR Critical Care [Title/Abstract] OR Critical Illness [Title/Abstract]))
(2) Embase	“vitamin d”/exp OR “25 hydroxyvitamin d”/exp OR “colecalciferol derivative”/exp OR “calcifediol”/exp OR “ergocalciferol derivative”/exp OR “vitamin d”:ab,ti OR “25 hydroxyvitamin d”:ab,ti OR “colecalciferol derivative”:ab,ti OR “calcifediol”:ab,ti OR “ergocalciferol derivative”:ab,ti AND (“newborn intensive care”/exp OR “pediatric intensive care unit”/exp OR “neonatal intensive care unit”/exp OR “critically ill”/exp OR “newborn intensive care”:ab,ti OR “pediatric intensive care unit”:ab,ti OR “neonatal intensive care unit”:ab,ti OR “critically ill”:ab,ti)
(3) Cochrane	(MeSH descriptor: [Vitamin D] explode all trees OR (Vitamin D OR Cholecalciferol OR 25 hydroxyvitamin D OR Hydroxycholecalciferols OR Calcifediol OR Calcitriol OR Dihydroxycholecalciferols OR Ergocalciferols):ti,ab,kw) AND (MeSH descriptor: [Critical Care] explode all trees OR MeSH descriptor: [Intensive Care Units] explode all trees OR MeSH descriptor: [Critical Illness] explode all trees OR (intensive care unit OR paediatric intensive care OR neonatal intensive care OR critical care OR critical illness):ti,ab,kw)
(4) Web of Science	(TS = (“Vitamin D*” OR Cholecalciferol OR “25 hydroxyvitamin D” OR Hydroxycholecalciferols OR Calcifediol OR Calcitriol OR Dihydroxycholecalciferols OR Ergocalciferols)) AND TS = (“intensive care unit*” OR “paediatric intensive care” OR “neonatal intensive care” OR “critical care” OR “critical illness”)

### Study Selection

Two independent reviewers (N.T., B.Y.) conducted the search. After removing duplicates, publications were initially screened based on their titles and abstracts. Full-text articles were then retrieved for detailed examination. Disagreements between the reviewers were resolved through discussion and a thorough review by a blinded independent third reviewer (D.C.). Studies were considered eligible if they included pediatric patients, specifically infants and children aged 1 month to 18 years, admitted to the PICU. To qualify, patients must have had their 25-hydroxyvitamin D levels measured at the time of admission as a baseline, allowing for subsequent categorization into a vitamin D-deficient state (<20 ng/mL) or a vitamin D-sufficient state (≥20 ng/mL) for further analysis [21]. A further subgroup analysis was performed for studies using a higher threshold for vitamin D status (ie, VDD < 30 ng/mL and vitamin D sufficiency [VDS] ≥ 30 ng/mL).

### Data Extraction

After identifying eligible studies, 2 reviewers (N.T., B.Y.) independently conducted data extraction using a standardized data collection form. Extracted data included study characteristics, patient demographics, interventions (if applicable), clinical information, and outcome measures.

The primary outcomes assessed were the incidence of sepsis and all-cause mortality. The study focused on the incidence of sepsis diagnosed after PICU admission. Definitions of sepsis varied but generally included positive microbiological results, septic shock as defined by the International Pediatric Sepsis Consensus Conference [22], and evaluations using scoring systems such as the Pediatric Logistic Organ Dysfunction (PELOD) score and the Pediatric Sequential Organ Failure Assessment (p-SOFA) score. All-cause mortality encompassed mortality within various timeframes, such as in-hospital mortality, and 28-, 60-, and 90-day mortality. Secondary outcomes included LOS, the need for and duration of MV, and the need for inotropic support.

### Quality Assessment

The risk of bias for observational studies was assessed using the Newcastle–Ottawa Quality Assessment Scale [23] (Table 2). These studies were evaluated based on several criteria, including the representativeness and selection of cohorts or cases, ascertainment of exposure, comparability of cohorts, assessment of outcomes, and adequacy of follow-up. Studies were rated as good, fair, or poor quality based on their performance across the domains of selection, comparability, and outcomes [45].

**Table 2.** Risk of bias summary for included study (Newcastle–Ottawa quality assessment scale)

Study	Selection				Comparability	Exposure			Outcomes			Total
	Representativeness of the exposed cohort	Selection of the nonexposed cohort	Ascertainment of exposure	Demonstration that outcome of interest was not present at start of study	Comparability of cohorts on the basis of the design or analysis	Ascertainment of exposure	Same method of ascertainment for cases and controls	Nonresponse rate	Assessment of outcome	Was follow-up long enough for outcomes to occur	Adequacy of follow-up of cohorts	
Loni et al (2023) [24]	1	1	1	1	1	NA	NA	NA	1	1	1	8
Ayulo et al (2014) [25]	1	1	1	1	1	NA	NA	NA	1	1	1	8
Rauniyar et al (2023) [26]	1	1	1	1	1	NA	NA	NA	1	1	1	8
Onwuneme et al (2015) [27]	1	1	1	1	2	NA	NA	NA	1	1	1	9
Ponnarmeni et al (2016) [28]	1	1	1	1	2	NA	NA	NA	1	1	1	9
Aşılıoğlu et al (2017) [18]	1	1	1	1	1	NA	NA	NA	1	1	1	8
Dang et al (2020) [29]	1	1	1	1	1	NA	NA	NA	1	1	1	8
Wang et al (2020) [30]	1	1	1	1	1	NA	NA	NA	1	1	1	8
Damke et al (2021) [31]	1	1	1	1	1	NA	NA	NA	1	1	1	8
Nurnaningsih et al (2018) [32]	1	1	1	1	1	NA	NA	NA	1	1	1	8
Qureshi et al (2022) [33]	1	1	1	1	1	NA	NA	NA	1	1	1	8
Sankar et al (2019) [34]	1	1	1	1	1	NA	NA	NA	1	1	1	8
Sankar et al (2016) [35]	1	1	1	1	1	NA	NA	NA	1	1	1	8
Jhang et al (2020) [36]	1	1	1	1	1	NA	NA	NA	1	1	1	8
Shah et al (2016) [37]	1	1	1	1	1	NA	NA	NA	1	1	1	8
Kumar et al (2020) [12]	1	1	1	1	1	NA	NA	NA	1	1	1	8
Prasad et al (2015) [38]	1	1	1	1	2	NA	NA	NA	1	1	1	9
Rey et al (2014) [39]	1	1	1	1	1	NA	NA	NA	1	1	1	8
Bustos et al (2016) [1]	1	1	1	1	1	NA	NA	NA	1	1	1	8
Ebenezer et al (2016) [4]	1	1	1	1	2	NA	NA	NA	1	1	1	9
Korwutthikulrangsri et al (2015) [40]	1	1	1	1	1	NA	NA	NA	1	1	1	8
García-Soler et al (2017) [19]	1	1	1	1	1	NA	NA	NA	1	1	1	8
Beyaz et al (2022) [41]	1	1	1	1	1	NA	NA	NA	1	1	1	8
Bansal et al (2022) [42]	1	1	1	1	1	NA	NA	NA	1	1	1	8
Dohain et al (2020) [43]	1	1	1	1	2	NA	NA	NA	1	1	1	9
Kubsad et al (2021) [44]	1	1	1	1	1	NA	NA	NA	1	1	1	8

**Figure 1.**
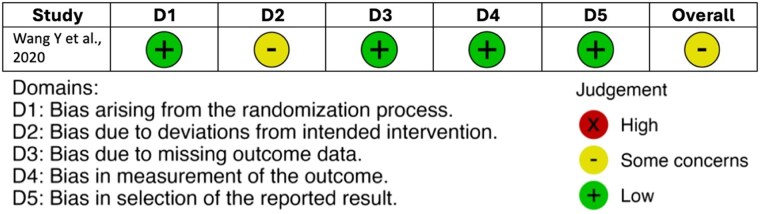
GRADE risk of bias tool for randomized controlled trials.

For RCTs, quality was assessed using the Grading of Recommendations Assessment, Development, and Evaluation (GRADE) Risk of Bias version 2 (GRADE RoB2) tool (Fig. 1). The trials were evaluated based on their randomization process, deviations from intended interventions, missing outcome data, measurement of outcomes, and selection of reported results. An overall risk of bias grade was then assigned, categorizing each study as having a low risk, some concerns, or high risk of bias [46].

All assessments were performed independently by 2 reviewers (N.T., B.Y.). Disagreements regarding methodological quality were resolved through adjudication by a third independent reviewer (D.C.).

### Statistical Analysis

All statistical analyses were performed using R statistical software. Pooled estimates were obtained using a random-effects model. Binary outcomes, such as all-cause mortality, incidence of sepsis, need for MV, and vasopressor support, were reported as odds ratio (OR) with 95% CI. Continuous outcomes, including duration of MV and LOS, were reported as mean differences with 95% CI. The proportion of between-study heterogeneity was assessed using the Cochran Q test and the I^2^ statistic.

## Results

### Study Selection

**Figure 2.**
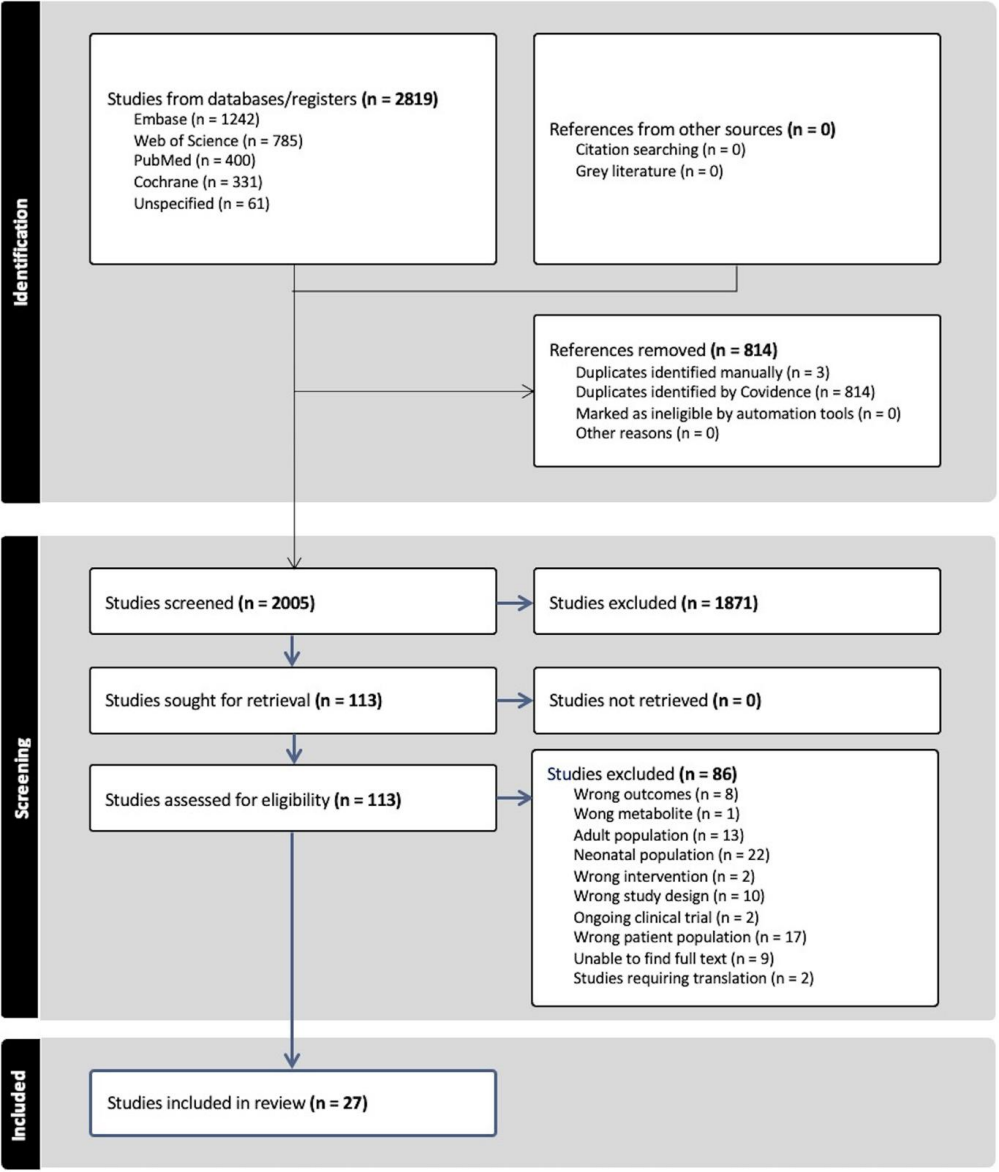
Selection process for eligible studies according to preferred reporting items for systematic reviews and meta-analyses.

The search was conducted in accordance with the Preferred Reporting Items for Systematic Reviews and Meta-Analyses (PRISMA) guidelines [47] (Fig. 2). The search yielded a total of 2819 nonduplicate titles and abstracts. Of these, 27 studies met the inclusion criteria for this review (Fig. 2). Table 3 provides a summary of the characteristics of the included studies. There were 26 observational studies, including 20 prospective cohort studies [1, 4, 12, 19, 24-26, 29, 31-35, 37-42, 48], 4 retrospective cohort studies [18, 36, 41, 44], and 2 case–control studies [1, 27]. Additionally, 1 RCT was included [30].

**Table 3.** Characteristics of studies on vitamin D levels and clinical outcomes in critically ill children

Study	Continent	Country	Country Income	Type of study	Total sample size	Vitamin D deficiency	Vitamin D sufficiency	Main admitting diagnosis	Gender (% male)	Age Range (months)	Intervention	Primary outcome reported	
												Mortality	Sepsis
Rey et al (2014) [39]	Europe	Spain	High	Observational Prospective	156	110	46	Respiratory, postoperative and infection	59.6	12-144	N	N	N
Ayulo et al (2014) [25]	North America	United States	High	Observational Prospective	216	155	61	Medical and postoperative	45	12-204	N	Y	N
Onwuneme et al (2015) [27]	Europe	Ireland	High	Case–control study	120	49	71	Respiratory	66	<144	N	N	Y
Prasad et al (2015) [38]	Asia	India	Low–Middle	Observational prospective	80	13	67	Medical	68.8	2-144	N	N	Y
Korwutthikulrangsri et al (2015) [40]	Asia	Thailand	Upper middle	Observational prospective	32	7	25	Sepsis and respiratory	50	12-144	N	Y	Y
Ponnarmeni et al (2016) [28]	Asia	India	Low–Middle	Case–control studies	124	61	63	Sepsis	64.5	12-144	N	Y	N
Sankar et al (2016) [35]	Asia	India	Low–Middle	Observational prospective	101	26	75	Sepsis and respiratory	52	1-204	N	Y	N
Bustos et al (2016) [1]	South America	United States	High	Observational prospective	90	51	39	Neurology	57	—	N	Y	N
Ebenezer et al (2016) [4]	Asia	India	Low–Middle	Observational prospective	52	31	21	Respiratory and shock	59.6	<12-156	N	Y	N
Shah et al (2016) [37]	Asia	India	Low-–Middle	Observational prospective	154	26	128	Respiratory and infection	66.2	6-102	N	Y	Y
García-Soler et al 2017 [19]	Europe	Spain	High	Observational prospective	340	191	149	Postoperative, noncardiac	54.4	6-192	N	Y	N
Aşılıoğlu et al (2017) [18]	Europe	Turkey	Upper middle	Observational retrospective	205	85	120	Underlying illness and respiratory	59.5	1-216	N	Y	N
Nurnaningsih and Rusmawatiningtyas (2018) [32]	Asia	Indonesia	Low–Middle	Observational prospective	42	19	23	Sepsis	65.2	1-168	N	Y	N
Sankar et al (2019) [34]	—	—	—	Observational prospective	43	12	31	Respiratory, gastrointestinal	56	<204	N	Y	N
Dang et al (2020) [29]	Asia	China	Upper middle	Observational prospective	296	180	116	Shock and respiratory	57.8	1-168	N	Y	Y
Jhang et al (2020) [36]	Asia	Korea	High	Observational retrospective	172	60	112	Respiratory	50	<216	N	Y	N
Kumar et al (2020) [12]	Asia	India	Low–Middle	Observational prospective	522	369	153	Medical	54.6	1-144	N	Y	N
Dohain et al (2020) [43]	Asia	Saudi Arabia	High	Observational prospective	69	35	34	Postoperative cardiac	59.4	1-90	N	Y	N
Wang et al (2020) [30]	Asia	China	Upper Middle	Randomized controlled trial	109	55	54	Respiratory and neurology	60	<168	Y	Y	N
Kubsad et al (2021) [44]	India	India	Low–middle	Observational Retrospective	84	19	65	Sepsis – respiratory	—	6-120	N	Y	N
Kumar et al (2021) [48]	Asia	India	Low–Middle	Observational prospective	384	209	175	Respiratory and neurology	54	12-168	N	N	N
Damke et al (2021) [31]	Asia	India	Low–Middle	Observational prospective	63	35	28	Cardiac	50.8	1-204	N	N	N
Qureshi et al (2022) [33]	Asia	Pakistan	Low–Middle	Observational prospective	782	594	188	—	50	1-180	N	Y	N
Beyaz et al (2022) [41]	Asia	India	Upper Middle	Observational retrospective	97	42	55	Respiratory and neurology	47	1-204	N	Y	Y
Bansal et al (2022) [42]	Asia	India	Low–Middle	Observational prospective	125	35	90	Medical	42	2-168	N	Y	N
Loni et al (2023) [24]	Asia	Bahrain	High	Observational prospective	119	63	56	Respiratory and neurology	60	1-168	N	Y	N
Rauniyar et al (2023) [26]	Asia	Nepal	Low–Middle	Observational prospective	105	31	74	Sepsis	51.1	1-180	N	N	N

### Study Characteristics

The included studies comprised a total of 4682 patients, with 45.2% (n = 2116) patients identified as vitamin D deficient. The gender distribution was largely balanced across studies, with males accounting for 54.6% (n = 2557) of patients. The age of patients ranged from 1 month to 18 years, with a pooled mean age of 46.9 months (95% CI 40.6-53.2). Eight studies were conducted in high-income countries [1, 19, 24, 25, 27, 36, 39, 43], while the remaining studies took place in upper middle- and lower middle-income countries, based on the World Bank's classification [49]. More specifically, 11 studies originated from India [4, 12, 28, 31, 35, 37, 38, 41, 42, 44, 48] and 2 studies were from Spain [19, 39], China [29, 30], and United States [1, 25] respectively. The remaining studies each came from Turkey [18], Ireland [27], Indonesia [32], Thailand [40], Korea [36], Saudi Arabia [43], Pakistan [33], Bahrain [24], and Nepal [26]. The country of origin was unspecified in 1 study [34]. Admitting diagnoses to the PICU were reported in 22 studies. The most common diagnoses included respiratory conditions in 16 studies, sepsis or infectious conditions in 6 studies, and neurological conditions in 5 studies.

## Primary Outcomes

### All-Cause Mortality

**Figure 3.**
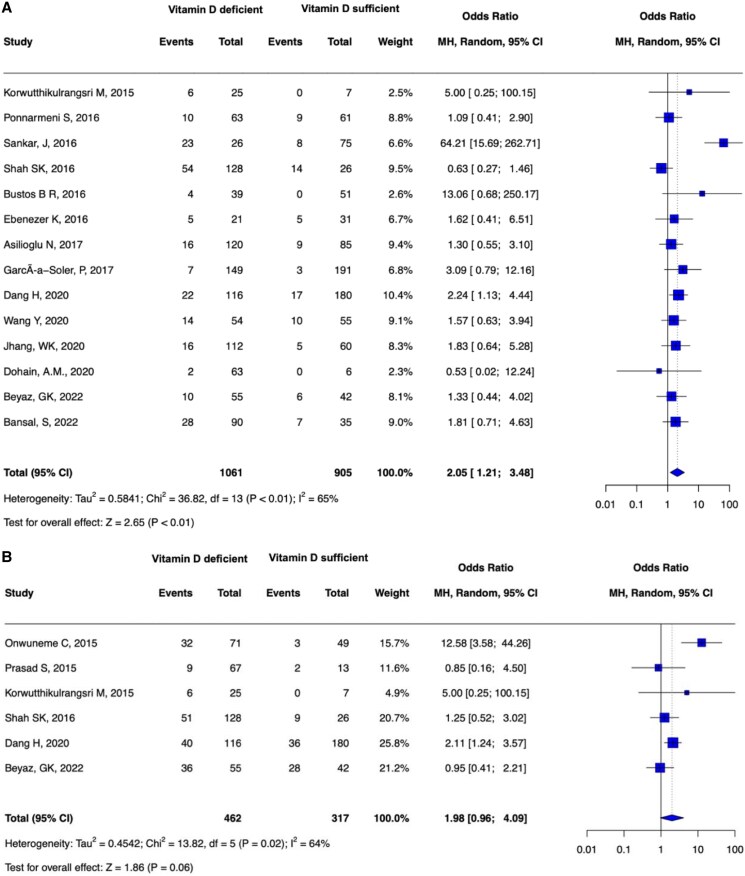
(A) All-cause mortality in patients with VDD in comparison to patients with VDS. (B) Incidence of sepsis in patients with VDD in comparison to patients with VDS.

A total of 21 studies [1, 4, 12, 18, 19, 24, 25, 28-30, 32-37, 40-43, 48] reported all-cause mortality, encompassing 3676 patients. Among these, 14 studies [1, 4, 18, 19, 28-30, 35-37, 40-43] categorized patients (n = 1966) into VDD (< 20 ng/mL) and VDS (≥ 20 ng/mL) groups. In these 14 studies, critically ill children with VDD had significantly higher odds of mortality (pooled OR 2.05, 95% CI 1.21-3.48) than those with VDS (Fig. 3A). The proportion of between-study heterogeneity was substantial across the included studies (I^2^ = 65%, *P* < .01).

### Sepsis

Six studies [27, 29, 37, 38, 40, 41] involving 884 patients examined the association between VDD and the incidence of sepsis following PICU admission. VDD was not significantly associated with increased odds of developing sepsis compared with patients with VDS (pooled OR 1.98, 95% CI 0.96-4.09) (Fig. 3B). The proportion of between-study heterogeneity was substantial among these studies (I^2^ = 64%, *P* = .06).

### Publication Bias

**Figure 4.**
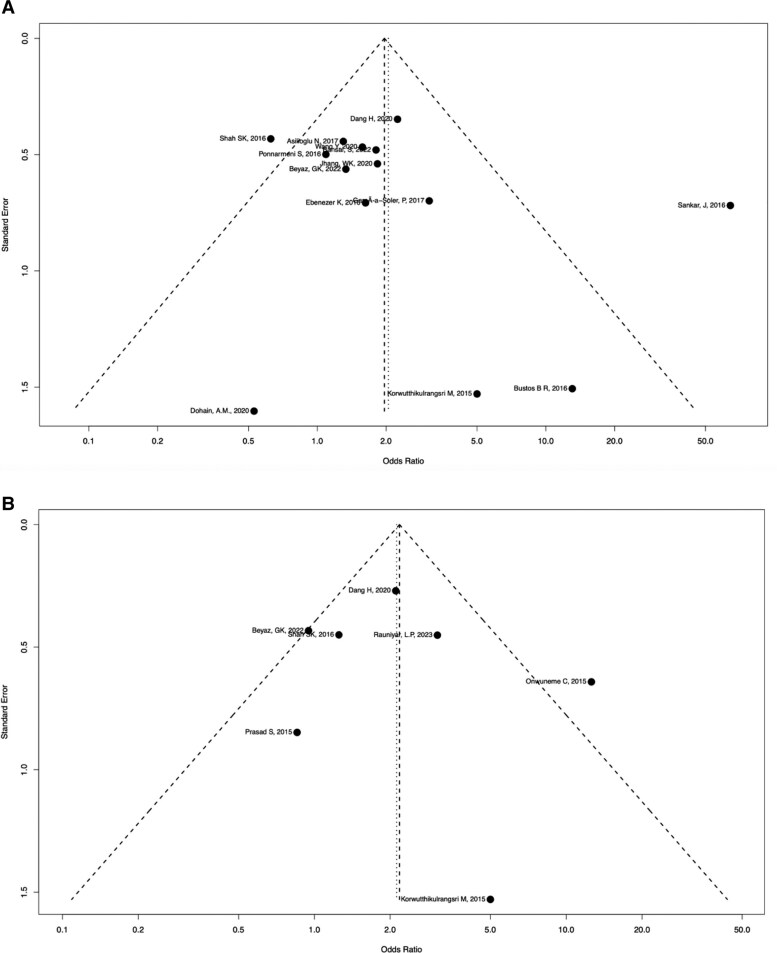
(A) Funnel plot for all-cause mortality. (B) Funnel plot for sepsis.

Funnel plots for the primary outcomes are presented in Fig. 4A and 4B. There was no evidence of publication bias in studies examining sepsis and all-cause mortality. For all-cause mortality, the Egger’s test yielded a *P* value of .261, indicating no significant publication bias. However, due to the limited number of studies on sepsis, Egger’s test could not be performed for this outcome.

## Secondary Outcomes

### Need for PICU Support

#### Inotrope use

Eleven studies [18, 19, 27, 28, 31, 35, 36, 39, 40, 42, 43] involving 1507 patients reported the need for inotropic support as a clinical outcome. The odds of requiring inotropic support were higher among critically ill pediatric patients with VDD (pooled OR 2.02, 95% CI 1.43-2.85) than in those without VDD. The proportion of between-study heterogeneity across these studies was moderate (I^2^ = 39%, *P* < .01).

#### Need for and duration of MV

Twelve studies [18, 24, 28, 29, 34-39, 41, 42] involving 1672 patients reported the need for MV. Ten studies [18, 28, 29, 35-39, 41, 42] classified patients into VDD and VDS groups. VDD was not associated with increased odds of requiring MV compared with VDS (pooled OR 1.02, 95% CI 0.68-1.54), with a moderate proportion of between-study heterogeneity (I^2^ = 63%, *P* < .01).

Six studies [24, 28, 35-37, 43] involving 408 patients reported the duration of MV. There was no significant difference in MV duration between VDD and VDS patients (mean difference = 0.35 days, 95% CI −1.66-2.35 days), with a low proportion of between-study heterogeneity (I^2^ = 13%, *P* = .33).

### Hospitalization Length of Stay

Fourteen studies [1, 18, 19, 24, 27, 28, 30, 34-37, 39, 40, 43] involving 1834 patients reported in-hospital LOS. Twelve studies [1, 18, 19, 27, 28, 30, 35-37, 39, 40, 43] classified patients into VDD and VDS groups. There was no significant difference in LOS between VDD and VDS patients (mean difference = 0.99 days, 95% CI −0.15-2.13 days), with a high proportion of between-study heterogeneity (I^2^ = 85%, *P* < .01).

### Studies With Higher Thresholds for Vitamin D Sufficiency

**Figure 5.**
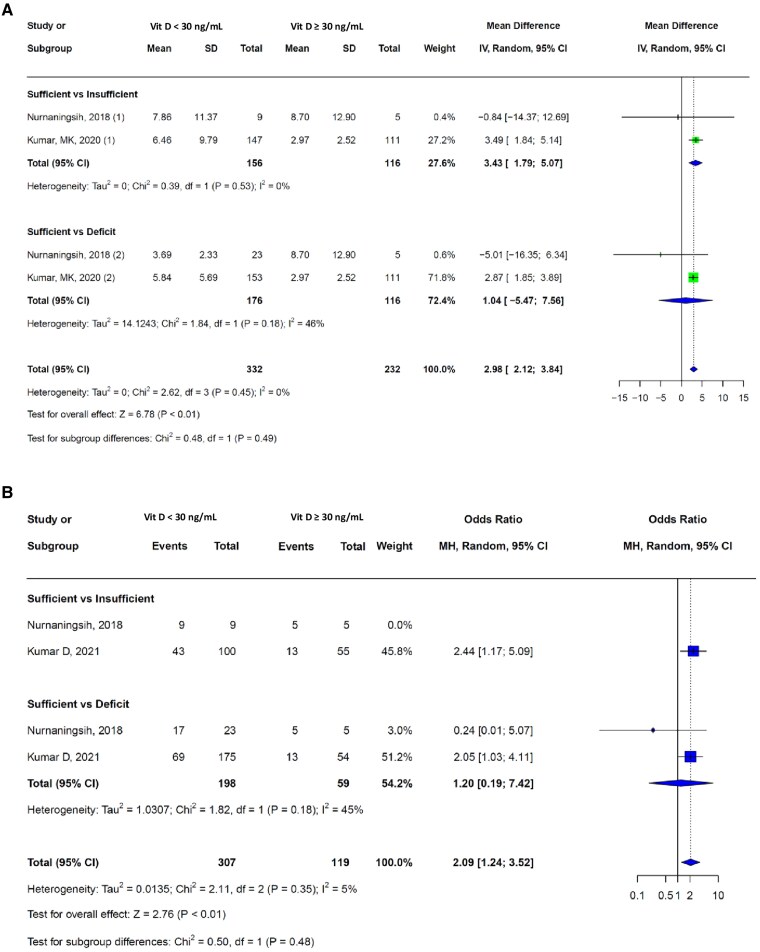
(A) Hospital length of stay in 2 studies which used higher threshold for VDD. (B) Need for mechanical ventilation in 2 studies which used higher threshold for VDD.

Three studies [12, 32, 48] used a higher threshold for VDS, defining it as 25-hydroxyvitamin D levels >30 ng/mL. In a subgroup analysis, participants with 25-hydroxyvitamin D levels >30 ng/mL were compared with those with levels between 20 and 29.9 ng/mL and <20 ng/mL. Patients with 25-hydroxyvitamin D levels <30 ng/mL had a significantly longer hospital stay (pooled mean difference = 2.98 days, 95% CI 2.12-3.84 days), with a low proportion of between-study heterogeneity (I^2^ = 0%, *P* = .45) (Fig. 5A). These patients also had higher odds of requiring MV (pooled OR 2.09, 95% CI: 1.24-3.52) than those with levels >30 ng/mL (Fig. 5B).

#### Risk of bias

The single RCT included was assessed as having “some concerns” in overall bias using the Risk of Bias 2 (RoB 2) tool [50] (Fig. 1). All the observational studies were evaluated to be of good quality according to the Newcastle–Ottawa Quality Assessment Scale [45] (Table 2).

## Discussion

In this meta-analysis of 27 studies involving a total of 4682 critically ill children, VDD was associated with increased all-cause mortality and the need for inotropic support. However, there was no significant association with new-onset sepsis after PICU admission, LOS, or the need for and duration of MV.

Multiple studies have reported conflicting findings on the association between VDD and clinical outcomes in critically ill children. Our results align with a prior systematic review and meta-analysis conducted in 2015 [16], which also identified an association between VDD and increased mortality (OR 1.62, 95% CI 1.11-2.36) and higher odds of requiring inotropic support (OR 1.97, 95% CI 1.49-2.61). However, unlike that prior meta-analysis, we did not find significantly increased odds of MV or infections. This discrepancy could be attributed to differences in the definition of sepsis across studies. While we focused on sepsis diagnosed after PICU admission, based on microbiological results or objective diagnostic criteria, the 2015 review included studies with a broader definition of sepsis, encompassing diagnoses up to 7 days before PICU admission and cases with “suspected” infections or extended empirical antibiotic use [1, 51]. Another meta-analysis of 13 observational studies in 2019 found a higher prevalence of VDD in children with sepsis, regardless of whether sepsis was diagnosed upon or after PICU admission [52]. These differences could be attributed to our study's focus on sepsis diagnosed specifically after PICU admission.

The diverse roles of vitamin D in various physiological pathways may underlie its association with clinical outcomes [53]. Vitamin D has been shown to modulate systemic inflammatory cytokines, such as tumor necrosis factor-α and interleukin-6, contributing to immune dysregulation in deficiency states [54]. It also supports the induction of T-regulatory cells, which suppress cytotoxic cells and limit immune reactivity during critical illness [55]. Additionally, vitamin D influences cardiovascular health by regulating myocardial hypertrophy, arterial compliance, and the renin–angiotensin–aldosterone system [56, 57]. These mechanisms may explain why VDD is prevalent in patients with heart failure and has been identified as an independent predictor of increased mortality in this population [58, 59]. Although these findings primarily originate from adult studies, pediatric research suggests similar associations. For instance, a cohort study of children with chronic kidney disease found that disruptions in calcium and phosphorus metabolism due to VDD were linked to increased left ventricular mass and disease progression [60]. These insights may elucidate the increased need for inotropic support in critically ill children with VDD, as demonstrated in our study and other systematic reviews [3, 16].

The strengths of our study include the use of subgroup analyses based on higher thresholds for 25-hydroxyvitamin D levels, allowing for a more nuanced understanding of VDD's impact. The inclusion of a large number of participants (n = 4682) enhances the reliability and generalizability of our findings. Additionally, the absence of timeline restrictions in our literature search ensures a comprehensive and representative summary of vitamin D's associations with critical care outcomes.

However, several limitations should be considered when interpreting our findings. First, the definitions of VDD varied across studies, resulting in multiple subgroups with differing thresholds, complicating direct comparisons and accurate estimation of associations [61]. Additionally, varying criteria for clinical outcomes like sepsis and mortality across countries and institutions may have influenced comparability. Moreover, while serum 25-hydroxyvitamin D levels are commonly used to define VDD, they may be inaccurate in critically ill patients due to the influence of vitamin D binding protein, an acute phase reactant affected by inflammation [62]. Many of the included studies were also observational in nature, limiting our ability to infer causation. Finally, the moderate to large degree of between-study heterogeneity makes it challenging to accurately interpret the underlying mechanisms driving the observed association between VDD and critical care outcomes, underscoring the need for further high-quality studies to examine this relationship further.

While our findings indicate an association between VDD and poorer critical care outcomes in PICU patients, most studies conducted in this area have been observational in nature. RCTs remain scarce, and even fewer have provided definitive evidence on the strength and direction of this association. Overall, summative data from our systematic review strongly suggests that well-designed RCTs are needed to investigate the impact of vitamin D replacement on clinical outcomes in critically ill children. Future trials should be conducted in institutions where 25-hydroxyvitamin D levels can be reliably assayed, with standardized laboratory practices and categorizations of vitamin D status aligned with contemporary consensus guidelines [21, 63]. High-dose vitamin D supplementation represents a promising intervention for safe and efficacious correction of VDD, with potential to improve clinical outcomes in children [64]. For instance, a RCT conducted in 2020 demonstrated that administering 150 000 IU of vitamin D to children with sepsis and VDD reduced the incidence of septic shock, although it did not affect LOS, MV duration, or mortality [30]. This may be attributed to the trial's sample size, which was based on a 40% increase in 25-hydroxyvitamin D levels as the minimally clinically important difference, rather than improvements in clinical outcomes. Recruiting larger sample sizes across multiple centers and basing sample size calculations on minimally clinically important differences in clinical outcomes could yield more robust findings and potentially influence future clinical practice in pediatric critical care.

In conclusion, our systematic review identified associations between VDD and several clinical outcomes in critically ill pediatric patients, including all-cause mortality and the need for inotropic support. However, no significant associations were found between VDD and sepsis after PICU admission, LOS, or the need for and duration of MV. Future RCTs are essential to establish definitive causal relationships and explore the potential benefits of vitamin D supplementation in this patient population.

## Data Availability

Original data generated and analyzed during this study are included in this published article or in the data repositories listed in References.

## Abbreviations

LOS: length of stay
MV: mechanical ventilation
PICU: Pediatric Intensive Care Unit
RCT: randomized controlled trial
VDD: vitamin D deficiency
VDS: vitamin D sufficiency
